# The Effect of Sulphuric Acid on the Thermostabilisation of Lignin/Biopolyamide 1010 Precursor Fibres for Carbon Fibre Production

**DOI:** 10.3390/molecules31152607

**Published:** 2026-07-26

**Authors:** Yi Zhang, Muhammed Hajee, A. Richard Horrocks, Baljinder Kandola

**Affiliations:** Institute for Materials Research and Innovation (IMRI), University of Greater Manchester, Deane Road, Bolton BL3 5AB, UKa.r.horrocks@greatermanchester.ac.uk (A.R.H.)

**Keywords:** lignin, biopolyamide, carbon fibre precursor, thermostabilisation, sulphuric acid, sulphonation

## Abstract

Sustainable precursor fibres for low-cost carbon fibre production are being extensively explored, particularly those based on lignin that are melt-blended with other thermoplastic polymers. Being prone to melting during melt processing is one of the main issues of using lignin to replace petroleum-based polyacrylonitrile (PAN) as precursor fibres. A time-consuming thermostabilisation process is normally needed to convert the thermoplastic blend’s polymer molecular chains to thermally stable structures prior to carbonisation. In order to accelerate the thermostabilisation process to facilitate industrial carbon fibre manufacturing, promising suitable surface treatments of lignin-based precursor fibres have emerged. In this work, sulphuric acid was used as a surface treatment agent to sensitise the crosslinking chemistry between interspersed components during the thermal stabilisation of lignin/polyamide blend precursor fibres. The impact of sulphuric acid concentration and the accelerated thermostabilisation conditions on the chemical structure and mechanical properties of precursor fibres were investigated. The surface-treated precursor fibres were directly stabilised at a high temperature, i.e., 200 °C, without a slow temperature elevation stage and the fusion of fibres using sulphuric acid with a concentration as low as 0.5 M. This was attributed to the improved condensation and crosslinking process via the sulphonation of lignin and to some extent PA1010. The isothermal time (<5 h) and final temperature (<240 °C) of the thermostabilisation process had significant effects on the final properties of surface-treated precursor fibres. The mechanical properties of thermostabilised precursor fibres were also investigated. The tensile modulus of the thermally stabilised fibres derived from surface-treated precursor fibres was increased by up to 50% using an optimised stabilisation process; however, tensile strength was decreased due to crosslinking.

## 1. Introduction

The significant increase in demand for carbon fibres based on the replacement of petroleum-based precursor fibres such as PAN by low-cost and environment-friendly biopolymers is reflected in the large numbers of research papers published in the past decade [1,2,3,4,5]. Lignin, the second most abundant biopolymer after cellulose and the first among aromatic biopolymers, has gained significant attention in terms of replacing petroleum-based polyacrylonitrile (PAN) on account of its low cost and renewable resources. Moreover, the large numbers of benzene rings and high carbon content (60–65%) in lignin molecules make lignin a promising candidate for carbon fibre production [5,6]. Thermostabilisation, also called thermal oxidative stabilisation, is one of the most important steps in producing carbon fibres in that the process converts the thermally fusible molecular structure of precursor fibres (PFs) into a thermally stable molecular structure, which prevents fusion when subsequently exposed to high temperatures during the carbonisation stage [7,8]. For lignin, the oxidation process is necessary to introduce oxygen to promote condensation and hence crosslinking, which is crucial to inhibiting or preventing the melting and subsequent fusion of the fibres during high-temperature carbonisation [9,10].

**Figure 1:**
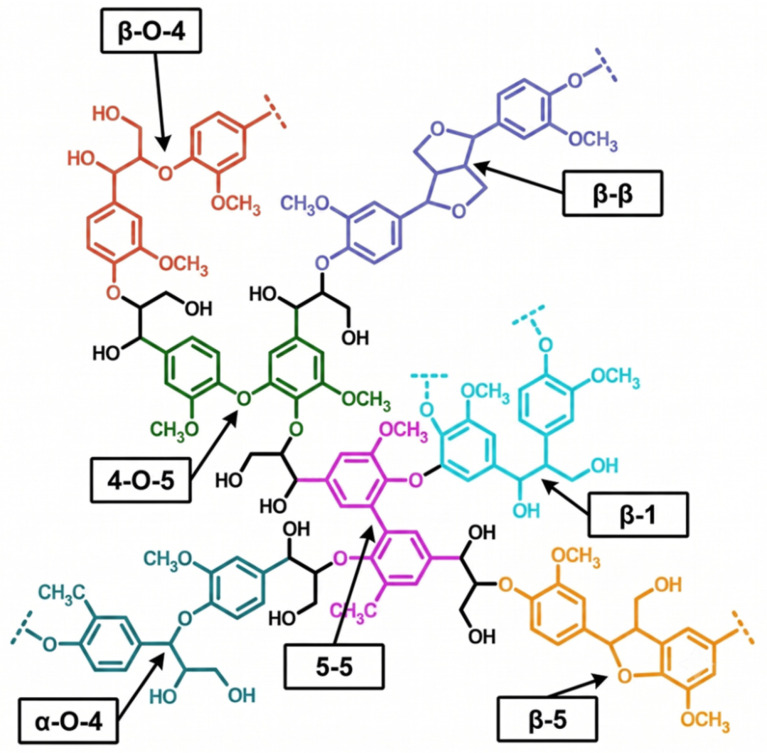
Major interunit linkages in lignin molecules. (Image adapted from ref. [11]. Copyright © 2010 American Chemical Society.)

However, the thermostabilisation of lignin-based PFs encounters many challenges due to the complex molecular structure of lignin, which consists of three main phenylpropane units: syringyl (S), guaiacyl (G) and p-hydroxyphenyl (H). These units are covalently combined to form highly branched 3D molecules with various interunit linkages, such as β-O-4, β-β, 4-O-5, β-1, 5-5, α-O-4 and β-5, as shown in Figure 1 [11]. The relative ratios of S, G and H units differ between hardwood and softwood lignins and vary depending on their botanical origin. The typical G/S ratios in hardwood are 1:1–1:3, whereas softwood lignin is composed of ~95% G units [12,13]. Since the C3 and C5 positions of G units are occupied by methoxy groups, the formation of linear structures in softwood lignin, which is dominated by G units, is less favourable than that in hardwood lignin, making it difficult to extrude into filaments, though with suitable chemical treatment, melt spinning would be possible [14]. Hence, hardwood is usually used for preparing carbon fibre precursors [13,15,16]. Similarly to polyacrylonitrile (PAN)-based fibres, oxidative stabilisation in air is the most widely used stabilisation method for lignin fibres. During oxidative thermal stabilisation, condensation between adjacent lignin units leads to the formation of a three-dimensional crosslinked infusible structure [10]. The thermostabilisation of lignin involves multiple procedures, such as the homolysis of C–O bonds, demethylation, the cleavages of C–C bonds, oxygenation, radical formation and the rearrangement of radicals [17]. It is commonly accepted that the early stage of the decomposition of lignin consists of the breakage of β-O-4 linkages, which refers to the cleavage of C–O bonds. Since G units contain two methoxy groups, they are likely to form condensed structures at the C5 position [11,13], resulting in oxidative condensation during thermal treatment [18]. Softwood lignin hence undergoes thermal stabilisation faster than hardwood lignin [19]. Hardwood lignins, with a higher number of S units, are less likely to undergo oxidative crosslinking; therefore, PFs made from hardwood lignin are prone to fusion during thermostabilisation, unless a very slow heating rate (≤0.2 °C [20]) is used or lignin is chemically modified.

In order to improve the processibility of lignin for precursor fibre production, the thermoplasticity and ductility of lignin need to be optimised. Blending with easily melt-processable polymers which are compatible with lignin is a low-cost and effective approach. In recent studies, lignin-based PFs have been produced from blends of lignin and synthetic and/or natural polymers, such as polyamide (PA) [21], polyurethane (PU) [22], polyethylene glycol (PEG) [23,24], poly(ethylene terephthalate) (PET) [25], high-density polyethylene (HDPE) [26] and cellulose [4,27,28], to improve the melt spinning behaviour of lignin. More recently, Muthuraj et al. [15,16] blended polyamides synthesised from biologically derived monomers (bio-PAs) such as PA1010, PA1012 and PA11 with hydroxypropyl-modified organosolv hardwood lignin (TcC) and organosolv hardwood lignin (TcA) using different combinations and ratios of these materials to improve the otherwise poor thermal stability and spinnability of each lignin. With a lignin/bio-PA ratio of 50/50 wt.%, the blends could be melt-spun into filaments with a tensile strength ≥ 20 MPa and moduli ≥ 500 MPa. The presence of lignin reduced the melting point of each PA, suggesting that the selected PAs and lignin were compatible. In order to improve the compatibility of TcC and PA1010, which showed the most promise in carbon fibre production, Muthuraj et al. [29] subsequently employed compatibilisers, i.e., ethylene–acrylic ester–maleic anhydride (MA) and ethylene–methyl acrylate–glycidyl methacrylate (GMA), to enhance compatibility between TcC and PA1010 by a reaction between hydroxyl groups with maleic anhydride groups in the MA compatibiliser or epoxy groups in the GMA compatibiliser via nucleophilic substitution. Although the tensile strengths and moduli of the final carbonised fibres were slightly lower than those of the uncompatibilised analogues due to insufficient tension applied during thermostabilisation and carbonisation, this method showed the potential benefit of using compatibilisers for carbon fibre production. However, the thermostabilisation process was time-consuming in that the process typically took 36 h, with heating rates ranging from 0.25 °C/min to 0.75 °C/min, which is not ideal for scaling up for industrial production [29,30].

Since the majority of lignin-based PFs need to be thermostabilised using a slow heating rate to increase *Tg*, reduce thermoplasticity and thus prevent melting during stabilisation, i.e., typically <0.5 °C/min to avoid fusion [27,29,30,31,32], the need for the acceleration of the thermostabilisation process is significant. In this respect, the surface treatment of PFs has shown great potential. For instance, Le et al. [33] reported that the time needed for the thermostabilisation of lignin/cellulose PFs treated with boric acid was reduced by 25% with a reduction in fibre fusion, and char yield was significantly increased compared to untreated PFs. Concentrated hydrochloric acid (HCl) solutions have also been used to modify the surface of lignin/PEG PFs to eliminate fusion before thermostabilisation [13]. Sulphonation treatment using sulphuric acid was reported by Goulis et al. [26]. In their work, lignin/HDPE PFs were immersed in sulphuric acid (96%) at 110 °C for 5 h and then 150 °C for 1 h, which were found to be optimised parameters for these specific PFs. The char yield of the sulphonated PFs was about 65%. The strong sulphonate bonds which were formed during sulphonation along HDPE chains and between HDPE and lignin were believed to be the main reason for extensive crosslinking.

In our recent work [34], we demonstrated that the surface treatment of 50:50 wt% lignin/bio-PA blended precursor fibres with a graphene oxide (GO) suspension facilitated thermal stabilisation at a significantly faster heating rate of 20 °C/min, compared to the typical 0.1–0.25 °C/min typically required for lignin-based fibres. The GO used in this study was synthesised via a modified Hummers method, employing 1 M sodium sulphate solution and 97% sulphuric acid as an electrolyte, with two carbon fibre mats serving as electrodes. Building on this work and inspired by Goulis et al. [26] on the sulphonation of lignin/HDPE fibres, here we investigate the sulphuric acid surface treatment of lignin/bio-PA blended PF to determine its role in the crosslinking reaction and to establish whether sulphuric acid treatment alone is sufficient to achieve the thermostabilisation acceleration previously observed with GO treatment [34]. This approach aims to eliminate the need for a more complex GO-based process, thereby reducing the time and energy consumed.

## 2. Results and Discussion

A series of H_2_SO_4_ solutions with different concentrations ranging from 0.1 M to 1.0 M was used to treat the PFs, and then the surface-treated PFs were heated at 85 °C for 1 h. After drying, these fibres were characterised in terms of the effects of sulphonation through an analysis of their physico-chemical and morphological changes.

### 2.1. Characterisation of PF for Sulphonation

**Figure 2:**
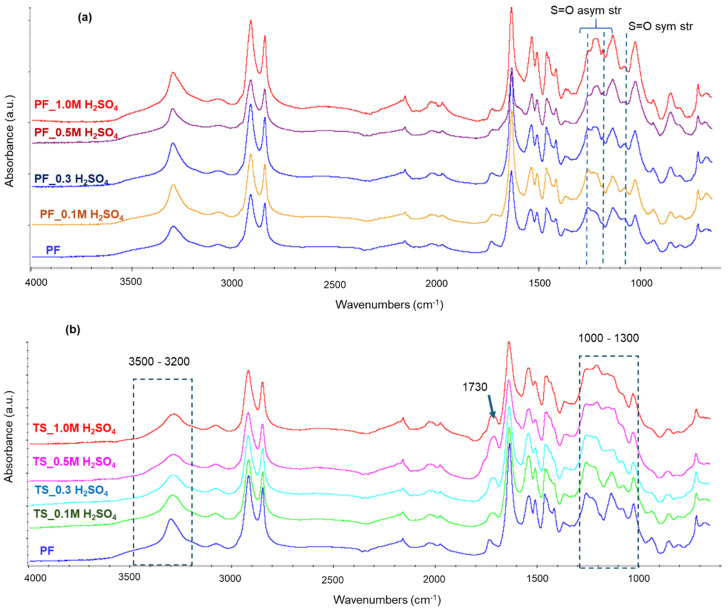
FTIR spectra of PFs (**a**) after surface treatment with sulphuric acid solutions and (**b**) after thermostabilisation at 200 °C for 1 h and 240 °C for 2 h.

The ATR-FTIR spectrum of the TcC/PA1010 fibres shown in Figure 2a and previously analysed in detail [16,30] displays characteristic peaks of lignin at 2920 cm^−1^ (C–H str.), 1740 cm^−1^ (C=O str.), 1235 cm^−1^ (ether groups), and 2851 and 2923 cm^−1^ (aromatic methoxyl groups), and that of PA1010 shows peaks at 1540 cm^−1^ (Amide II: N–H bend., C–N str.), 1640 cm^−1^ (Amide I: C=O str.), and 3302 cm^−1^ (O–H and/or N–H str.). The new bands at 1267, 1190 (S=O asymmetric str.) and 1075 cm^−1^ (S=O symmetric str.) in the spectra of surface-treated TcC/PA1010 with H_2_SO_4_ in Figure 2a confirmed the insertion of -SO_3_H groups into the polymer chains within the blended PFs. The increase in the intensity of these signals in the region with an increasing concentration of H_2_SO_4_ is related to the increase in the number of sulphonic acid groups (-SO_3_H).

**Figure 3:**
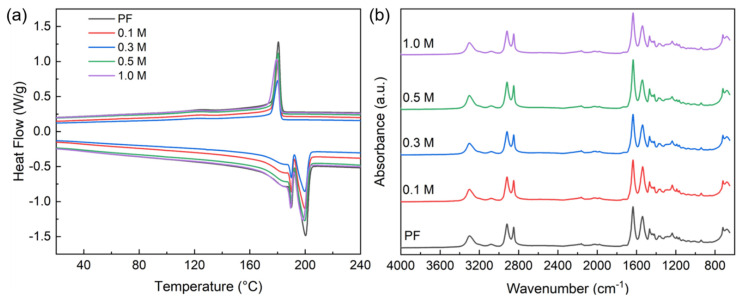
DCS curves (**a**) and FTIR spectra (**b**) of PA1010 fibres after surface treatment with sulphuric acid solutions.

In order to understand whether only lignin or both the lignin and PA1010 components of the blend were sulphonated, PA1010 filaments were also treated with H_2_SO_4_ solutions of concentrations ranging from 0.1 M to 1.0 M, similarly to the PFs. The ATR-FTIR spectra of the treated filaments shown in Figure 3 indicated that generally, the sulphonation of the PA1010 molecules did not significantly affect the characteristic polyamide bands in Figure 3. At the relatively low acid concentrations used in this study, it might be expected that the sulphonation of lignin would be more likely since while the sulphonation of aliphatic nylons usually occurs at the active α-position of the amide group, it requires powerful agents such as fuming sulphuric acid [35], whereas hydroxy-alkylated lignins require the use of less powerful ones such as concentrated sulphuric acid or sodium sulphite [36].

Since melt-extruded filaments cannot be produced from lignin, they could not be treated with sulphuric acid and characterised in a similar way as the blended fibre or PA1010. However, as discussed above, the FTIR spectra of PA1010 fibres treated with acid solution did not show any characteristic peak of sulphonate groups; hence the effects observed in lignin–PA1010 can be attributed primarily due to lignin.

**Figure 4:**
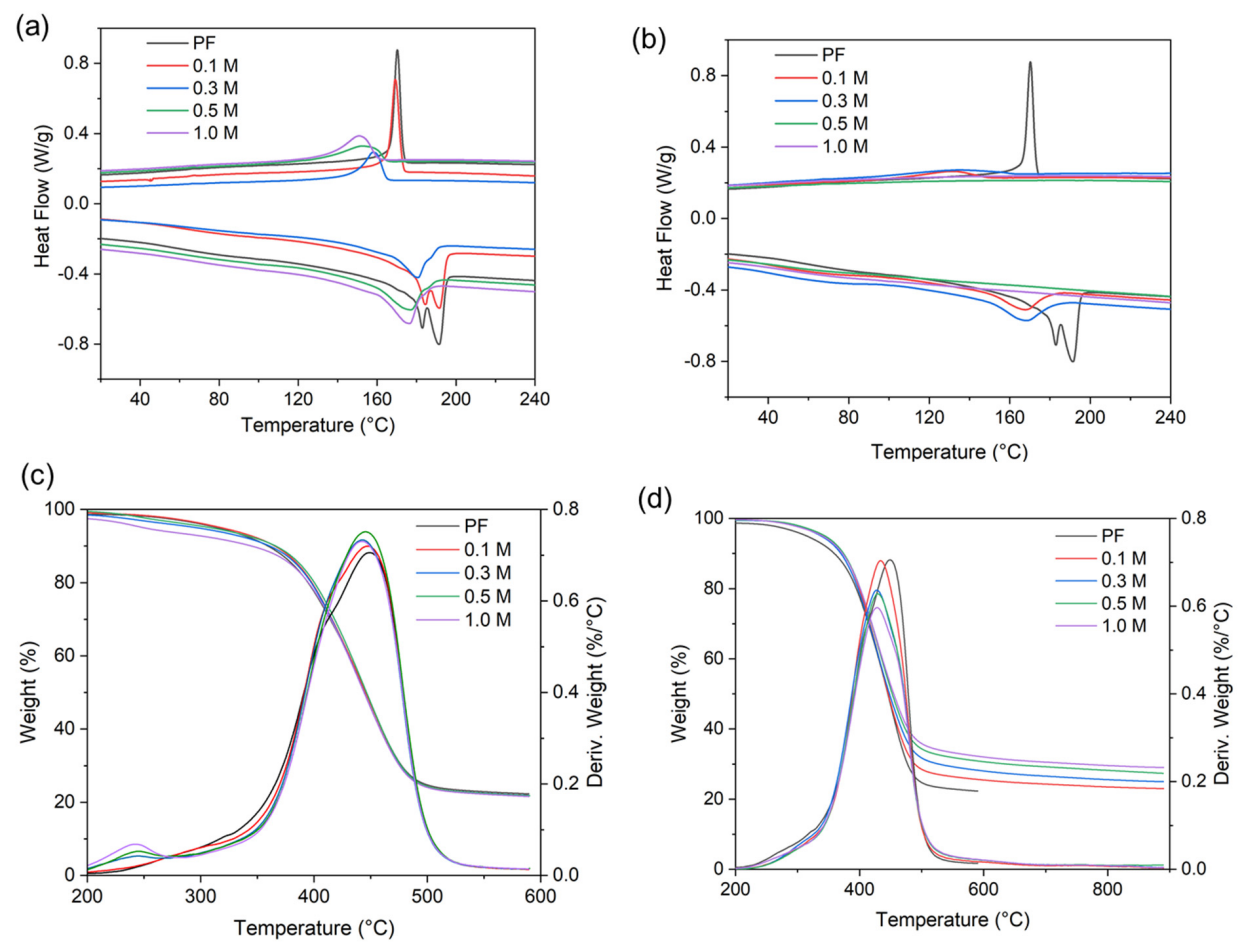
DSC (**a**,**b**) and TGA-DTG curves under nitrogen atmosphere (**c**,**d**) of PFs after surface treatment with sulphuric acid solutions (**a**,**c**) and after thermostabilisation (**b**,**d**) at 200 °C for 1 h and 240 °C for 2 h.

The thermophysical properties of the treated fibres were studied by DSC. From the DSC curves shown in Figure 4a, the untreated PF exhibited melting and recrystallisation regions, which can be attributed to the PA1010 component of the blend, as discussed in detail in previous publications [15,16,29,30]. The lignin phase, being amorphous, does not show any similar fusion and crystallisation behaviour. While these melting and recrystallisation peaks were still present in the treated fibres, indicating that the PFs retained thermoplasticity after surface treatment with H_2_SO_4_, their nature had changed, thus confirming that some degree of sulphonation of the PA1010 component occurred. With regard to fusion endotherms, no dual-melting peaks were observed in PFs treated with 0.3 M, 0.5 M and 1.0 M H_2_SO_4_ solutions. It has been reported that the multiple melting peaks of PA1010 are caused by the rearrangement of lamellae because polyamide crystals can be easily thickened by annealing. The low-temperature melting peak at 183 °C (see Table 1) is formed by melting thin lamellae, while the high-temperature one at 192 °C is formed by the melting of thickened lamellae [37,38]. The disappearance of the dual-melting peaks observed in 0.3, 0.5 and 1 M H_2_SO_4_-treated fibres is suggested to derive from the introduced -SO_3_H groups which would have hindered the rearrangement of PA chains to form thicker lamellae during heating and annealing. Although dual-melting peaks were observed in the 0.1 M-exposed PFs, the high-temperature melting peak was weaker compared to that of the untreated PFs. This is because the number of introduced -SO_3_H groups was not high enough to completely prevent the rearrangement of PA polymer chains to form thicker lamellae; therefore, dual-melting peaks in the 0.1 M group were still observed. Moreover, the introduced -SO_3_H groups shifted the onset of melting and the melting peak to a lower temperature (from 192 °C in untreated fibres to 174 °C in 1 M H_2_SO_4_-treated fibres). In essence, the more introduced -SO_3_H groups, the greater shifts observed. This is because the insertion of -SO_3_H groups hinders the formation of large crystals of PA1010, hence resulting in a lower onset melting temperature and melting peaks. The effect of the introduced -SO_3_H groups on crystallisation behaviour agrees with this proposal that the insertion of -SO_3_H groups restricts the mobility of PA1010 chains during cooling, which slows down crystallisation, hence resulting in lower crystallisation temperatures.

In conclusion, while there was no evidence of the sulphonation of the PA1010 component of the blend from the ATR-FTIR results, given the evidence of thermophysical changes in the DSC study, it is more likely that the sulphonation of both the PA and lignin phases occurred.

### 2.2. Effect of Concentration of H_2_SO_4_ on Thermostabilisation of PFs

As discussed in Section 1, PFs need to be transformed into an infusible thermoset-like structure during thermostabilisation before undergoing extreme high-temperature carbonisation. These fibres were thermally stabilised by heating them to 200 °C for 1 h and then 240 °C for 2 h using the procedure discussed in Section 3.3. While the untreated fibres could not withstand this treatment and fused during the process, all H_2_SO_4_-treated fibres could be thermostabilised. Oxidative stabilisation induces various chemical changes, such as oxidation, condensation, crosslinking, and cyclisation in PFs. Figure 2b and Figure 4b show the FTIR spectra and DSC curves of the surface-treated PFs after thermostabilisation, respectively.

As can be seen from Figure 4b and Table 1, thermostabilised fibres from PFs treated with 0.1 M and 0.3 M H_2_SO_4_ still have the melting endotherm of PA1010 present, indicating that these are not completely thermostabilised, whereas PFs treated with 0.5 M and 1 M H_2_SO_4_ are fully thermostabilised. This indicates that a minimum of 0.5 M H_2_SO_4_ for the treatment of PF is required for the latter to withstand accelerated thermostabilisation.

Along with an increase in H_2_SO_4_ concentration, the PFs became progressively dehydrated, as reflected by the continuous decrease in the intensity of the O–H stretching band (3500–3200 cm^−1^) in Figure 2b. In contrast, the intensity of the signal related to oxidation, i.e., C=O stretching (1730 cm^−1^) in unconjugated ketones, carbonyls and carboxyl groups, aldehydes and anhydrides, increased with H_2_SO_4_ concentration. These chemical changes during the conversion of lignin to carbon fibres have also been reported in the literature [39]. This study shows that the sulphonation of lignin accelerates these reactions. The region from 1000 to 1300 cm^−1^ relating to ethers and esters became broad without specific individual peaks observed. These broader regions related to higher H_2_SO_4_ concentrations, which have been observed in other studies [19,40], could be attributed to the increased crosslinking of intra- and/or inter-lignin molecules [39]. Therefore, it is suggested that sulphonation offers newly formed crosslinkable groups to facilitate the formation of conjugated structures of C=C, C=O and O=S=O via dehydrogenation, oxidation and other reactions to achieve extensive crosslinking.

**Figure 5:**
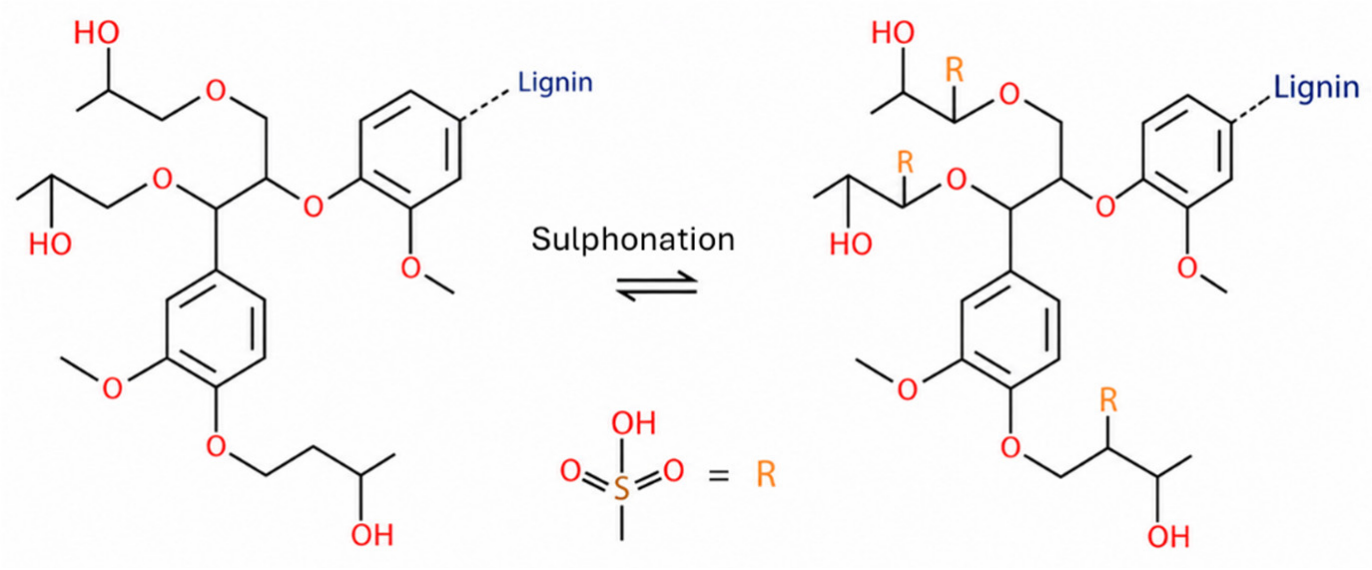
Proposed sulphonation of TcC lignin molecules.

It has also been previously reported that H_2_SO_4_ can produce SO_3_, H_3_O^+^ and HSO_4_^−^ through hydrogen–oxygen protonation, as shown in Equation (1) [26]. The released electrophile SO_3_ may then be attached to the lignin molecule, as postulated in Figure 5. Based on bond dissociation energies, initial hydrogen abstraction and hence sulphonation would be expected to occur at weaker benzylic and aliphatic C-H bonds rather than at the aromatic C–H position [41]. However, lignin is a complex heterogenous biopolymer (see Figure 1), any modification of which will involve multiple competing reactions [42], and so sulphonation reactions could be accompanied by other reactions which lead to eventual sulphonation, as evidenced by the FTIR spectra. Upon heating, sulphonic groups act as bridges between lignin molecules and/or lignin and hydrocarbon polymers to form crosslinks.

2H_2_SO_4_ ⇌ SO_3_ + H_3_O^+^ + HSO_4_^−^ (1)

**Figure 6:**
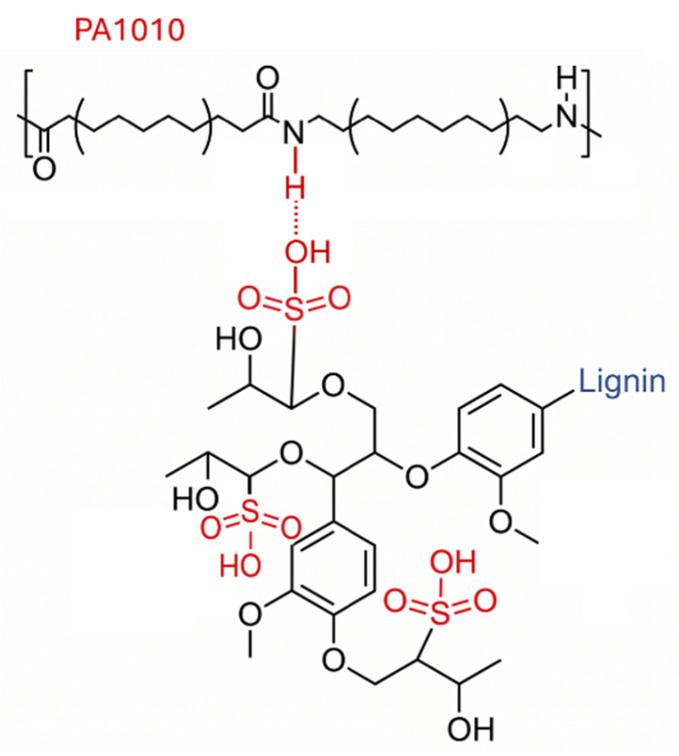
Chemical interaction between PA1010 and lignin.

Based on the above discussion, the chemical interaction between lignin and PA1010 through hydrogen bonding in the presence of sulphuric acid may be proposed, as illustrated in Figure 6. Subsequent reactions most likely follow, although further research would be necessary to elucidate their nature.

Thermogravimetric analysis (TGA) in a nitrogen atmosphere was used to further investigate this hypothesis of crosslinking occurring during the thermostabilisation stage. The TGA-DTG curves of the surface-treated PFs before and after thermostabilisation at 200 °C for 1 h and 240 °C for 2 h are shown in Figure 4c,d. All surface-untreated and -treated PFs had similar char yields before thermostabilisation (Figure 4c and Table 1). As can be seen from Figure 4c, the introduced sulphonic acid groups reduced the onset of degradation of the PFs. This is because heating acidic PFs catalyses the depolymerisation of the PA1010 component and hydrolyses hydroxypropyl-modified TcC, as well as promoting some degree of lignin dehydration. It would appear that these competing effects counterbalanced each other to yield unchanged char levels. However, sulphonated PFs after thermostabilisation showed higher char yields than the untreated PF analogues, as shown in Figure 4c and Table 1. Furthermore, char yield increased with the concentration of H_2_SO_4_ solutions, and the remaining mass under nitrogen at 900 °C increased from 23.2% to 29.6% as concentration increased from 0.1 M to 1.0 M compared to 22.0% in untreated PF. This would suggest that the presence of sulphuric acid promoted crosslinking in agreement with other research that has reported that sulphonation has a positive impact on char yield during the thermostabilisation of lignin [43].

**Figure 7:**
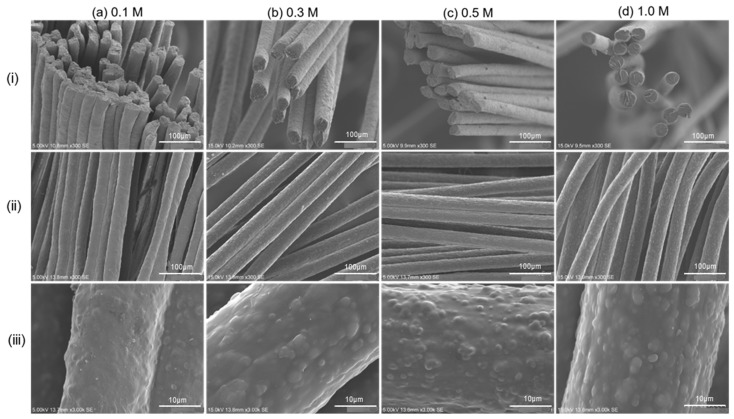
SEM images of (**a**) 0.1 M, (**b**) 0.3 M, (**c**) 0.5 M and (**d**) 1.0 M H_2_SO_4_-treated PFs after thermostabilisation, each shown at three different magnifications (i)–(iii).

The SEM images in Figure 7 revealed the morphology of the sulphonated PFs after thermostabilisation. Only the group of PFs treated with 0.1 M H_2_SO_4_ solution showed the presence of filament fusion after thermostabilisation (see Figure 7a), whilst no fusion was observed in the other groups, indicating that good shape retention was achieved by surface treatment using sulphuric acid (Figure 7b–d). Because of the plasticity and fusion in 0.1 M H_2_SO_4_-treated fibres, the diameter of the thermostabilised PFs was found to be about 20.1 µm, while the other thermally stabilised filaments had similar diameter ranges, i.e., 30.3–33.3 µm. The surface morphologies of the thermostabilised PFs showed negligible differences between all samples.

### 2.3. Effect of Isothermal Temperature and Time on Thermostabilisation of PFs

**Figure 8:**
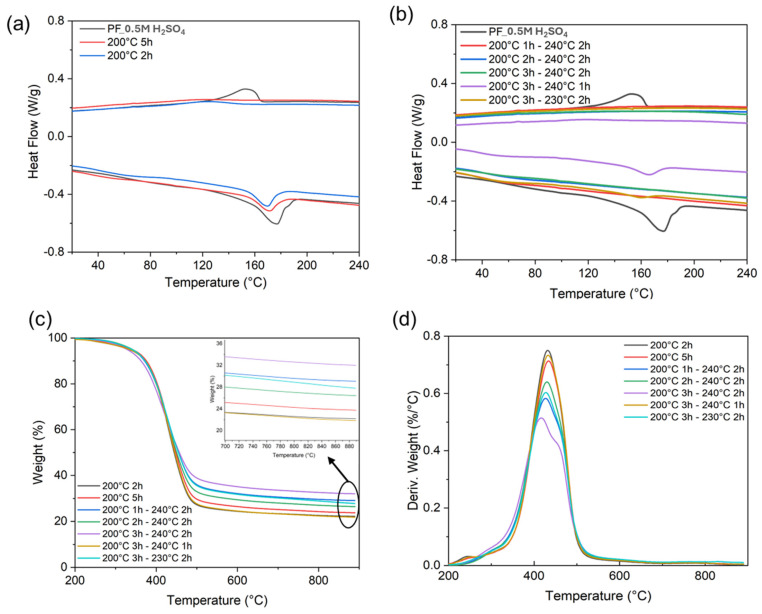
DSC (**a**,**b**) and TGA-DTG (**c**,**d**) curves of sulphonated PFs with 0.5 M H_2_SO_4_ and thermostabilised using different programmes.

Besides the concentration of H_2_SO_4_ solutions, the effect of the time and temperature used for thermostabilisation on the change in the functional groups of the PFs was investigated. Firstly, the PFs surface-treated with 0.5 M H_2_SO_4_ were stabilised at 200 °C for 2 h and 5 h to investigate the effect of isothermal time on the conversion of thermoplastic PFs into thermosets. Figure 8a shows that the surface-treated PFs which were stabilised at 200 °C for 2 h or 5 h still had DSC PA1010 melting peaks at 178–179 °C after thermostabilisation, indicating insufficient PA–lignin crosslinking within the PFs, which is not ideal for the subsequent carbonisation. In order to improve the level of crosslinking, two-stage isothermal processes were used to see how isothermal temperature and time affect the crosslinking process [30]. Various combinations of isothermal time and temperature were applied, as shown in Figure 8b. It can be seen that the temperature and time of the second stage were more important than those of the first stage, as melting peaks were only observed in groups where a shorter isothermal time (I h) and a lower temperature (230 °C) were used in the second stage. This result was similar to a previous study which found that the final stabilisation temperature was the most influential parameter on the properties of PFs [9]. In fibres where no melting peaks were observed, including the sample where an isothermal temperature of 240 °C for 2 h was used in the second stage, this indicated the presence of extensive PA–lignin crosslinking, which prevents the fusion of fibres during the subsequent carbonisation stage.

**Figure 9:**
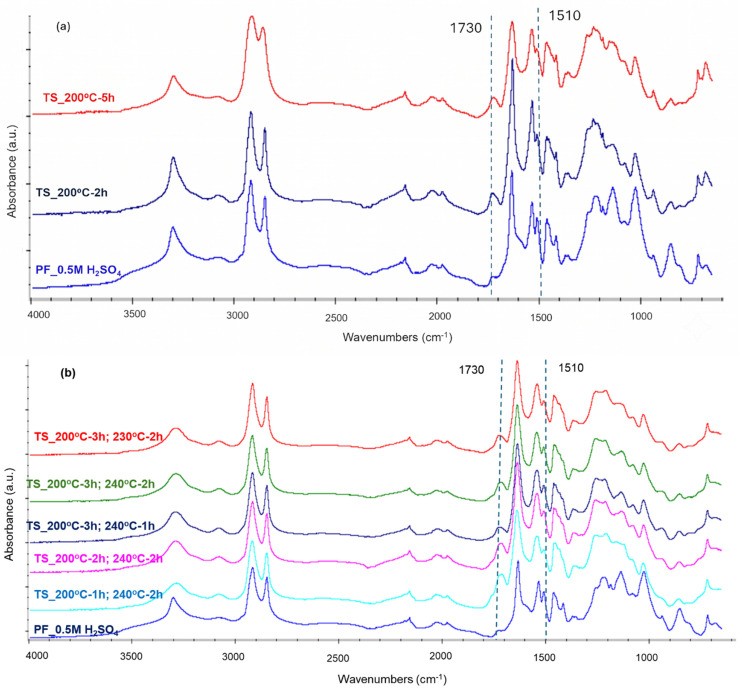
FTIR spectra of PFs sulphonated with 0.5 M H_2_SO_4_ and thermostabilised using different isothermal temperatures and times. (**a**) Single-stage; (**b**) dual-stage.

Figure 9 shows the FTIR spectra of the functional group changes within sulphonated PFs after various thermostabilisation procedures. At the earlier stages (lower temperatures) of stabilisation, free radicals may be produced by the cleavage of ether bonds including arylglycerol-β-O-4 aryl ether linkages, as other carbon–carbon major linkages, such as β–β, β-5, β-1, and 5–5, are more resistant to degradation [18,20]. The chemical reaction of free radicals in the presence of oxygen introduced ketones, carbonyl, and carboxyl groups which are highly crosslinkable [40]. These changes in functional groups were reflected by the increase in the signal around 1730 cm^−1^, as shown in Figure 9a,b. As temperature increased, crosslinking would most probably occur by the insertion of these groups into unsaturated carbons, such as ester and anhydride linkages in lignin molecules. The specific changes in functional groups induced by stabilisation are reflected in the gradual disappearance of the characteristic lignin signal at 1510 cm^−1^ (aromatic skeletal vibrations), suggesting that their initial structure was degraded.

TGA confirmed the increased crosslinking level by showing an increased char yield, as shown in Figure 8c and Table 2. Char yield increased with isothermal time and temperature. Sulphonated PFs, which were thermostabilised at 200 °C for 3 h and then at 240 °C for 2 h showed the highest char yield of 32.6%. The PFs stabilised without the second stage and shorter second stage showed the lowest three char yields. This result again agrees with the above hypothesis that a higher isothermal temperature and time benefit crosslinking and hence result in a higher char yield. The DTG curves in Figure 8d also show that the less crosslinked groups decomposed faster than groups which were extensively crosslinked. Hence, the optimum condition for the maximum crosslinking of the thermostabilisation of 0.5 M sulphuric acid-treated fibres is a two-stage isothermal process comprising 200 °C for 3 h and then 240 °C for 2 h.

**Figure 10:**
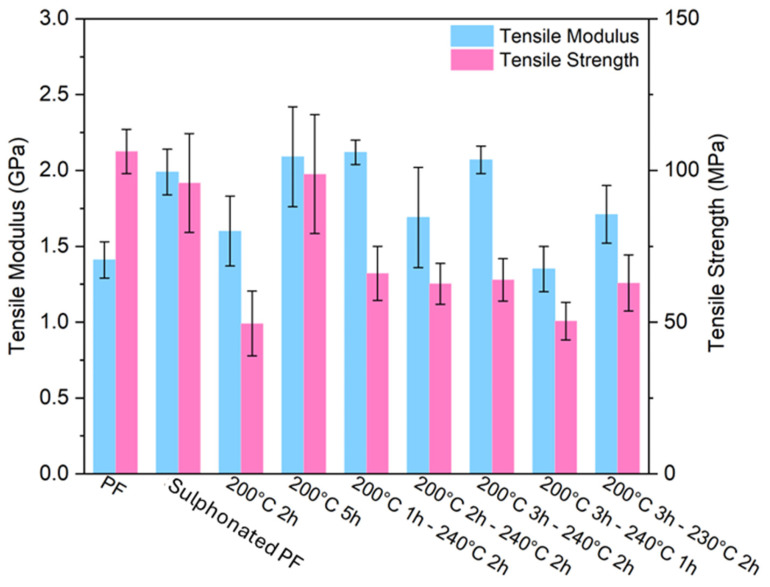
Tensile properties of PFs in different stages.

Figure 10 compares the tensile strengths and moduli of PFs both unstabilised and thermally stabilised under various single- and double-stage conditions. The sulphonation of PF helped in increasing the modulus of PF from 1.41 ± 0.12 GPa to 1.99 ± 0.15 GPa, whereas strength was not affected (Figure 10), indicating that H_2_SO_4_ treatment stiffens the fibre but makes it weaker. In general, after the thermal stabilisation of the sulphonated fibres, the tensile modulus of most of them remained similar to or decreased (1.35 ± 0.15–2.12 ± 0.08 GP) compared to that of the sulphonated precursor fibre (1.99 ± 0.15 GP), while strength decreased under all conditions. This indicates that the sulphonation of the PFs had already initiated crosslinking, enabling the fibres to withstand the very high heating rate employed in this work. And the subsequent heating during thermal stabilisation helped maintain or further increase fibre stiffness. In terms of isothermal temperature and time, after one-stage thermal stabilisation, both the modulus and strength of the sulphonated PF decreased at 200 °C for 2 h to 1.60 ± 0.23 GPa and 50 ± 0.11 MPa. This observation is consistent with the DSC results, which showed that a PA1010 melting endotherm remained present (Figure 8a), indicating that the fibres had undergone insufficient stabilisation. However, increasing the time to 5 h restored both properties. In the two-stage process, while clear trends cannot be established because of the relatively large error range, these results support the finding from DSC that the temperature and time of the second stage have a greater influence on mechanical properties than those of the first stage. The isothermal time of 1 h in the first stage (at 200 °C) and 2 h in the second stage (at 240 °C) provided the optimal results in terms of both modulus (2.12 ± 0.08 GPa) and tensile strength (63 ± 7 MPa) values. Increasing the isothermal time of the first stage had little effect on fibre properties. Reducing the time of the second stage to 1 h (sample TS_200 °C-3 h; 240 °C-1 h) or temperature to 230 °C (sample TS_200 °C-1 h; 230 °C-2 h) decreased the tensile properties of the fibres, which supports the observation from the DSC results that the melting endotherm remained present in both of these samples, indicating insufficient stabilisation.

In our previous work, we extensively studied the effect of heating rates on the thermal stabilisation of TcC/PA1010 (without any surface treatment) [30]. For successful thermal stabiliastion, a two-step process had to be adapted as follows: (i) heating at a 0.25 °C/min heating rate to 180 °C, holding isothermally for 1 h, cooling to room temperature at 5 °C/min and (ii) heating again at a 0.25 °C/min heating rate to 250 °C and holding isothermally for 2 h before cooling back to room temperature at 5 °C/min [29,30], taking 29 h overall. Using a heating rate of 0.25 °C/min increased the tensile modulus of PF from 1.41 ± 0.12 GPa to 2.2 ± 0.11 GP [30]. However, the modulus decreased at 0.5 °C/min, while at 0.75 and 1 °C/min, considerable fibre fusion occurred. Although the fused fibres could be tested as a tow, they could no longer be tested as single filaments. With sulphuric acid treatment, thermal stabilisation could be accelerated, producing fibres with mechanical properties comparable to those of untreated fibres thermally stabilised using a much lower heating rate of 0.25 °C/min.

### 2.4. Carbonised Fibres

**Figure 11:**
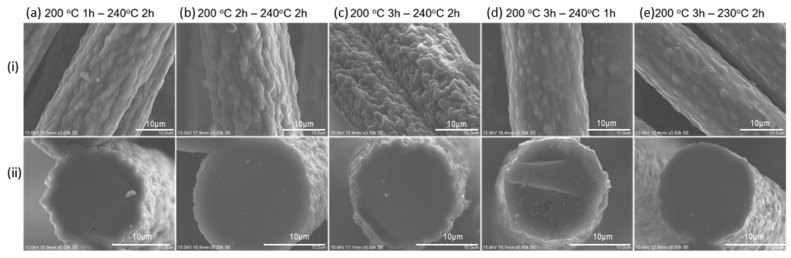
SEM images of the (i) surface and (ii) cross section of carbonised fibres thermostabilised with different parameters: (**a**) 200 °C-1 h, 240 °C-2 h, (**b**) 200 °C-2 h, 240 °C-2 h, (**c**) 200 °C-3 h, 240 °C-2 h, (**d**) 200 °C-3 h, 240 °C-1 h and (**e**) 200 °C-3 h, 230 °C-2 h.

Thermostabilised sulphonated fibres were carbonised at 1000 °C under nitrogen. Fibres thermostabilised at 200 °C for 2 and 5 h, which showed melting endotherms in DSC, fused together during carbonisation because of insufficient crosslinking; hence no separated single carbon fibres were obtained. All other fibres stabilised in the two-stage process could be successfully carbonised. The SEM images of these fibres in Figure 11 show that the fibres which were stabilised using the longest isothermal time in both stages and higher temperature in the second stage, i.e., 200 °C, 3 h and 240 °C, 2 h conditions (Figure 11c), showed significantly different morphologies compared to all others. The surfaces of these carbonised fibres were quite uneven, suggesting shrinkage following mass loss during carbonisation. The morphologies of the single-stage, thermally stabilised carbonised fibres in the first two groups in Figure 11 (fibres thermostabilised at 200 °C for 1 or 2 h and 240 °C for 2 h, Figure 11a,b) were similar, being less rough than the two-stage fibres thermostabilised at 200 °C for 3 h and 240 °C for 2 h (Figure 11c). When a lower final temperature and a shorter time length of the second stage were used for thermostabilisation, the surfaces of derived carbonised fibres (Figure 11d,e) appeared to be much smoother than those of the first three groups. However, the cross-sections of all fibres were relatively similar, with the absence of apparent voids and defects.

Unfortunately, the carbonised fibres in all groups were extremely brittle, and tensile testing could not be carried out as their handling was impossible. Although the sulphonated PFs obtained in this work did not melt during thermostabilisation and carbonisation, most likely, the lack of graphitic structure resulted in their brittleness.

In Table 3, the salient properties of the thermostabilised fibres pre-treated with 0.5 M sulphuric acid are compared with those of fibres treated with GO-electrolysed solution, as reported in our previous work [34]. In the former, lower first- and second-stage temperatures were used for thermostabilisation. While both sets of fibres could be successfully thermostabilised using their respective conditions (see Table 3), the char yield of the GO-electrolysed solution-treated fibres was much higher (39.3%) than that of the 0.5 M H_2_SO_4_-treated fibres (28.2%), indicating that carbon yield from the former would be significantly higher. The mechanical properties of the latter were also inferior to those of the former, indicating that while H_2_SO_4_ treatment provides sufficient crosslinking to withstand the accelerated thermal stabilisation process, the presence of GO further enhances crosslinking and most likely acts as a physical template to increase graphitisation during both thermostabilisation and subsequent carbonisation [44]. Therefore, while our previous research [34] demonstrated the potential of GO within a sulphuric acidic environment for the formation of carbon fibres from lignin–PA1010 precursor fibres, this work demonstrated that while sulphuric acid treatment alone has a positive effect on the crosslinking process, the presence of GO or any other graphitising material is essential if resilient carbon fibres are to be realised.

## 3. Experimental Section

### 3.1. Materials

Lignin/PA1010 precursor fibres (diameter: 40 ± 5 µm) were obtained by melt blending a hydroxypropyl-modified organosolv hardwood lignin (Tecnaro, Ilsfeld, Germany), TcC (M_w_ = 11,357 g/mol) and polyamide 1010 (PA1010, 3960R) (Mw = 120,540) at a 50:50 wt% ratio, and they were subsequently melt-spun by using a Spinmaster semi-industrial multi-filament extrusion line at Centexbel, Belgium, using previously described parameters [34]. Sulphuric acid (H_2_SO_4_, 98%) was sourced from Sigma-Aldrich, Gillingham, UK and distilled water was home-made.

### 3.2. Surface Treatment

Precursor fibres of 110 ± 5 mm length were wrapped on glass tubes (diameter: 5 mm; length: 90 mm) and immersed in 40 mL sulphuric acid solutions with different concentrations (0.1, 0.3, 0.5 and 1.0 M) at 85 °C for 1 h using a previously described setup [34]. The treated PFs were dried using tissue without washing; then the dried PFs were prepared for thermostabilisation.

### 3.3. Thermostabilisation

The surface-treated PFs were thermally stabilised using a temperature-controlled tube furnace (Pyrotherm Furnaces, UK), equipped with a quartz tube (2.5 cm diameter). Precursor filaments were horizontally mounted on a stainless-steel bar (300 mm × 30 mm × 3 mm) with two alumina pulleys spaced 70 cm apart. Filaments were fixed at one end with masking tape and tensioned (~2 g) at the other using hanging paper clips. The tensioned samples were then placed inside the furnace for thermal treatment. A detailed description of the experimental procedure can be found in reference [34]. Thermostabilisation was conducted using either a one- or two-stage process with different parameters under air atmospheric conditions. In the one-stage process, the PFs were isothermally thermostabilised at 200 °C for 2 and 5 h. In the two-stage thermostabilisation process, the filaments were isothermally stabilised at 200 °C for different time lengths ranging from 1 to 3 h, followed by increasing the temperature to either 230 °C or 240 °C at a heating rate of 10 °C/min and an isothermal hold for either 1 or 2 h.

### 3.4. Carbonisation

The stabilised fibres were carbonised using the same tube furnace that was employed for thermal stabilisation, operating under a nitrogen atmosphere. Thermally stabilised fibres, each approximately 20 cm in length, were held under tension within the quartz tube and securely fixed at both ends to a ceramic stage using a set of screws. Further experimental details are provided elsewhere [34]. The fibres were pre-heated at 50 °C with a nitrogen flow of 30 mL/min for 30 min prior to heating to 1000 °C with a heating rate of 20 °C/min and an isothermal hold at 1000 °C for 3 min, as per a previously optimised carbonisation procedure [30,34]. Subsequently, the carbonised samples were cooled down to room temperature.

### 3.5. Characterisation

The degree of oxidative crosslinking of the fibres was investigated using differential scanning calorimetry, DSC (Q2000 DSC, TA instruments, Wilmslow, UK). The changes in the thermal stability and char yield of the thermostabilised filaments were analysed by thermogravimetric analysis, TGA (SDQ-Q600, TA instruments). The detailed programmes used for DSC and TGA can be referred to in our previous study [29]. The morphologies of the surface-treated, thermostabilised and carbonised filaments were analysed using a Hitachi S-3400 N scanning electron microscope (SEM). The specimens were placed vertically in the SEM sample holder to observe their cross-section morphologies. The changes in the functional groups of the PFs before and after surface treatment and thermostabilisation were investigated using FTIR (Thermo Fisher Nicolet iS10, Waltham, MA, USA) in conjunction with an attenuated total reflection (ATR) adapter. The spectra were recorded from 400 to 4000 cm^−1^ with 4 cm^−1^ resolution and 32 consecutive scans. Each spectrum was normalised to C-H stretch absorption at 2923 cm^−1^, using the reference value from TcC/PA1010, to allow for a direct comparison of different spectra.

### 3.6. Tensile Properties of Fibres

The tensile properties of individual fibres were measured according to the BS ISO 11566:1996 standard [45] with an Instron model 3369 using a 100 N load cell at a cross-head speed of 5 mm/min with a gauge length of 25 mm. Fibre diameters were determined using SEM, and each fibre diameter measurement was an average of 25–30 individual measurements.

## 4. Conclusions

In this study, the accelerated thermal stabilisation of lignin/polyamide (TcC/PA1010) precursor fibres was achieved by pre-treating the fibres with sulphuric acid prior to thermal stabilisation. Immersing the fibres in 0.5 M sulphuric acid at 85 °C for 1 h could result in sulphonation, as evidenced by the presence of sulphonic groups on the fibre surface by Fourier Transform Infrared (FTIR) spectroscopy. It was proposed that the lignin component of the blend is mainly sulphonated. However, while there was no direct evidence of chemical interaction between sulphuric acid and PA1010 by FTIR, its thermal behaviour was modified in that DSC showed that the characteristic double endothermic melting peaks of PA1010 were replaced by a single, less intense peak upon acid treatment, with intensity decreasing as sulphuric acid concentration increased. This interaction facilitated crosslinking between the components of the blend, as evidenced by the increased tensile modulus of sulphonated fibres compared to untreated fibres. The crosslinking was further enhanced during the thermal stabilisation stage, enabling the fibres to withstand the very high heating rate (~20 °C/min) compared with the 0.1–0.25 °C/min range typically required for untreated lignin-based fibres. Thermal stabilisation was evidenced by the disappearance of the melting endotherm of PA1010 in the DSC curves of thermally stabilised fibres, along with an increased char yield observed in thermogravimetric analysis (TGA). Mechanical testing revealed that the tensile modulus of the stabilised fibres increased compared to untreated fibres but remained unchanged or slightly increased compared to sulphonated fibres, while tensile strength decreased. The thermally stabilised fibres were successfully carbonised, yielding void-free carbon fibres, which unfortunately were too brittle to test for their mechanical properties. Although the observed brittleness could be interpreted as a limitation of the present work in producing satisfactory carbon fibres, a comparison with our previous study employing GO electrolysis treatment demonstrates that sulphuric acid treatment alone is sufficient to promote crosslinking and achieve acceptable levels of thermal stabilisation. However, the results also clearly indicate that the presence of GO is essential for the formation of mechanically useful carbon fibres, most likely due to its graphitisation-templating effect. Accordingly, during sulphuric acid surface treatment, the incorporation of GO, or a comparable graphitising agent, is required to obtain high-quality carbon fibres.

## Figures and Tables

**Table 1:** Summary of DSC and TGA results of precursor fibres (PFs) after surface treatment with sulphuric acid solutions and after thermostabilisation (TS) at 200 °C for 1 h and 240 °C for 2 h.

Sample	DSC Melting Endotherm (°C)		TGA	
		T_Onset_ * (°C)	DTG Max (°C)	Char Residue (%) at 800 °C
PF (TcC/PA1010)	183, 192	304	450	22.0
PF_0.1 M H_2_SO_4_	184, 192	320	447	21.9
PF_0.3 M H_2_SO_4_	180	298	442	21.9
PF_0.5 M H_2_SO_4_	176	309	445	21.9
PF_1.0 M H_2_SO_4_	174	250	441	21.7
TS_0.1 M H_2_SO_4_	169	340	435	23.2
TS_0.3 M H_2_SO_4_	167	341	427	25.5
TS_0.5 M H_2_SO_4_	-	343	431	28.2
TS_1.0 M H_2_SO_4_	-	342	428	29.6

* T_Onset_ = Temperature at which 5% mass loss occurred.

**Table 2:** Summary of DSC and TGA results of thermostabilised precursor fibres (PFs) after surface treatment with sulphuric acid solutions, using different thermostabilisation programmes.

Sample	DSC Melting Endotherm (°C)	TGA		
		T_Onset_ * (°C)	DTG Max (°C)	Char Residue (%) at 800 °C
TS_200 °C-2 h	178	336	431	22.6
TS_200 °C-5 h	179	341	435	24.3
TS_200 °C-1 h; 240 °C-2 h	-	340	427	29.6
TS_200 °C-2 h; 240 °C-2 h	-	342	431	27
TS_200 °C-3 h; 240 °C-2 h	-	329	418	32.6
TS_200 °C-3 h; 240 °C-1 h	166	333	432	22.4
TS_200 °C-1 h; 230 °C-2 h	161	339	431	22.6

* T_Onset_ = Temperature at which 5% mass loss occurred.

**Table 3:** Comparison of thermal and mechanical properties of thermostabilised fibres pre-treated with 0.5 M sulphuric acid and with GO-electrolysed solution [b].

Sample	Thermostabilisation Conditions	TGA—Char Residue at 800 °C	Tensile Properties	
			Modulus (GPa)	Strength (MPa)
PF	-	22.0	1.41 ± 0.12	106 ± 7
GO-electrolysed sol-treated [34]	210 °C for 1 h; 275 °C for 1 h	39.3	2.30 ± 0.30	89 ± 3
Surface-treated with 0.5 M H_2_SO_4_	200 °C for 1 h; 240 °C for 2 h	28.2	1.69 ± 0.33	66 ± 17

## Data Availability

The data that support the findings of this study are available from the corresponding author upon request.

## References

[1] Wan L., Liu H., Yang Y., Dai L., Si C. (2025). Lignin-derived carbon fibers: A green path from biomass to advanced materials. Carbon Energy.

[2] Wang S., Bai J., Innocent M.T., Wang Q., Xiang H., Tang J., Zhu M. (2022). Lignin-based carbon fibers: Formation, modification and potential applications. Green Energy Environ..

[3] Sun S.-C., Xu Y., Wen J.-L., Yuan T.-Q., Sun R.-C. (2022). Recent advances in lignin-based carbon fibers (LCFs): Precursors, fabrications, properties, and applications. Green Chem..

[4] Byrne N., De Silva R., Ma Y., Sixta H., Hummel M. (2018). Enhanced stabilization of cellulose-lignin hybrid filaments for carbon fiber production. Cellulose.

[5] Souto F., Calado V., Pereira N. (2018). Lignin-based carbon fiber: A current overview. Mater. Res. Express.

[6] Bengtsson A., Bengtsson J., Sedin M., Sjöholm E. (2019). Carbon Fibers from Lignin-Cellulose Precursors: Effect of Stabilization Conditions. ACS Sustain. Chem. Eng..

[7] Huang X. (2009). Fabrication and Properties of Carbon Fibers. Materials.

[8] Ogale A.A., Zhang M., Jin J. (2016). Recent advances in carbon fibers derived from biobased precursors. J. Appl. Polym. Sci..

[9] Cho M., Ko F.K., Renneckar S. (2019). Impact of Thermal Oxidative Stabilization on the Performance of Lignin-Based Carbon Nanofiber Mats. ACS Omega.

[10] Brodin I., Sjöholm E., Gellerstedt G. (2010). The behavior of kraft lignin during thermal treatment. J. Anal. Appl. Pyrolysis.

[11] Zakzeski J., Bruijnincx P.C.A., Jongerius A.L., Weckhuysen B.M. (2010). The Catalytic Valorization of Lignin for the Production of Renewable Chemicals. Chem. Rev..

[12] Chatterjee S., Saito T. (2015). Lignin-Derived Advanced Carbon Materials. ChemSusChem.

[13] Lin J., Kubo S., Yamada T., Koda K., Uraki Y. (2012). Chemical thermostabilization for the preparation of carbon fibers from softwood lignin. BioResources.

[14] Veettil U.T., Moreno A., Huertas-Alonso A.J., Morsali M., Pylypchuk I.V., Liu L.-Y., Sipponen M.H. (2023). Mechanically recyclable melt-spun fibers from lignin esters and iron oxide nanoparticles: Towards circular lignin materials. Green Chem..

[15] Muthuraj R., Hajee M., Horrocks A., Kandola B.K. (2019). Biopolymer blends from hardwood lignin and bio-polyamides: Compatibility and miscibility. Int. J. Biol. Macromol..

[16] Muthuraj R., Horrocks A., Kandola B.K. (2020). Hydroxypropyl-modified and organosolv lignin/bio-based polyamide blend filaments as carbon fibre precursors. J. Mater. Sci..

[17] Akpan E.I., Adeosun S.O. (2019). Sustainable Lignin for Carbon Fibers: Principles, Techniques, and Applications.

[18] Fang W., Yang S., Wang X.-L., Yuan T.-Q., Sun R.-C. (2017). Manufacture and application of lignin-based carbon fibers (LCFs) and lignin-based carbon nanofibers (LCNFs). Green Chem..

[19] Norberg I., Nordström Y., Drougge R., Gellerstedt G., Sjöholm E. (2013). A new method for stabilizing softwood kraft lignin fibers for carbon fiber production. J. Appl. Polym. Sci..

[20] Braun J.L., Holtman K.M., Kadla J.F. (2005). Lignin-based carbon fibers: Oxidative thermostabilisation of kraft lignin. Carbon.

[21] Mandlekar N., Cayla A., Rault F., Giraud S., Salaün F., Malucelli G., Guan J. (2017). Thermal Stability and Fire Retardant Properties of Polyamide 11 Microcomposites Containing Different Lignins. Ind. Eng. Chem. Res..

[22] Culebras M., Beaucamp A., Wang Y., Clauss M.M., Frank E., Collins M.N. (2018). Biobased Structurally Compatible Polymer Blends Based on Lignin and Thermoplastic Elastomer Polyurethane as Carbon Fiber Precursors. ACS Sustain. Chem. Eng..

[23] Lin J., Koda K., Kubo S., Yamada T., Enoki M., Uraki Y. (2013). Improvement of Mechanical Properties of Softwood Lignin-Based Carbon Fibers. J. Wood Chem. Technol..

[24] Kadla J., Kubo S., Venditti R., Gilbert R., Compere A., Griffith W. (2002). Lignin-based carbon fibers for composite fiber applications. Carbon.

[25] Beaucamp A., Wang Y., Culebras M., Collins M.N. (2019). Carbon fibres from renewable resources: The role of the lignin molecular structure in its blendability with biobased poly(ethylene terephthalate). Green Chem..

[26] Goulis P., Konstantopoulos G., Kartsonakis I.A., Mpalias K., Anagnou S., Dragatogiannis D., Charitidis C. (2017). Thermal treatment of melt-spun fibers based on high density polyethylene and lignin. C.

[27] Cho M., Karaaslan M., Chowdhury S., Ko F., Renneckar S. (2018). Skipping Oxidative Thermal Stabilization for Lignin-Based Carbon Nanofibers. ACS Sustain. Chem. Eng..

[28] Trogen M., Le N.-D., Sawada D., Guizani C., Lourençon T.V., Pitkänen L., Sixta H., Shah R., O’Neill H., Balakshin M. (2021). Cellulose-lignin composite fibres as precursors for carbon fibres. Part 1—Manufacturing and properties of precursor fibres. Carbohydr. Polym..

[29] Muthuraj R., Hajee M., Horrocks A.R., Kandola B.K. (2021). Effect of compatibilizers on lignin/bio-polyamide blend carbon precursor filament properties and their potential for thermostabilisation and carbonisation. Polym. Test..

[30] Kandola B.K., Hajee M., Xiang A., Horrocks A.R. (2025). Lignin-based carbon fibres: Effect of bio-polyamide on oxidative thermal stabilisation of lignin. J. Mater. Sci. Technol..

[31] Hosseinaei O., Harper D.P., Bozell J.J., Rials T.G. (2017). Improving Processing and Performance of Pure Lignin Carbon Fibers through Hardwood and Herbaceous Lignin Blends. Int. J. Mol. Sci..

[32] Baker D.A., Gallego N.C., Baker F.S. (2012). On the characterization and spinning of an organic-purified lignin toward the manufacture of low-cost carbon fiber. J. Appl. Polym. Sci..

[33] Le N.-D., Trogen M., Varley R.J., Hummel M., Byrne N. (2021). Effect of boric acid on the stabilisation of cellulose-lignin filaments as precursors for carbon fibres. Cellulose.

[34] Kandola B.K., Hewage T.A.M., Hajee M., Horrocks A.R., Culebras M., Collins M.N. (2025). Biobased lignin/polyamide filaments surface–modified with electrochemically produced graphene oxide to improve their thermal stabilisation behaviour as precursors for carbon fibre production. Int. J. Biol. Macromol..

[35] Vaya J., Zilkha A. (1974). Sulfonation of polyamides. Isr. J. Chem..

[36] Gao W., Inwood J.P.W., Fatehi P. (2019). Sulfonation of hydroxymethylated lignin and Its application. J. Biores. Bioprod..

[37] Nishitani Y., Mukaida J., Yamanaka T., Kajiyama T., Kitano T. (2016). Thermal properties of hemp fiber filled polyamide 1010 biomass composites and the blend of these composites and polyamide 11 elastomer. AIP Conference Proceedings.

[38] Li L., Li C.Y., Ni C., Rong L., Hsiao B. (2007). Structure and crystallization behavior of Nylon 66/multi-walled carbon nanotube nanocomposites at low carbon nanotube contents. Polymer.

[39] Mainka H., Hilphert L., Busse S., Edelmann F., Haak E., Herrmann A.S. (2015). Characterization of the major reactions during conversion of lignin to carbon fiber. J. Mater. Res. Technol..

[40] Li Y., Cui D., Tong Y., Xu L. (2013). Study on structure and thermal stability properties of lignin during thermostabilisation and carbonization. Int. J. Biol. Macromol..

[41] Luo Y.-R. (2007). Comprehensive Handbook of Chemical Bond Energies.

[42] Laurichesse S., Avérous L. (2014). Chemical modification of lignins: Towards biobased polymers. Prog. Polym. Sci..

[43] Barton B.E., Patton J.T., Hukkanen E.J., Bernius M.T. (2017). Two-Step Sulfonation Process for the Conversion of Polymer Fibers to Carbon Fibers. U.S. Patent.

[44] Ike S.N., Vander Wal R.L. (2024). Improved Graphitization of Lignin by Templating Using Graphene Oxide Additives. ACS Appl. Bio Mater..

[45] (1996). Carbon Fibre—Determination of the Tensile Properties of Single-Filament Specimens.

